# Hendra Virus Infection Dynamics in Australian Fruit Bats

**DOI:** 10.1371/journal.pone.0028678

**Published:** 2011-12-09

**Authors:** Hume Field, Carol de Jong, Deb Melville, Craig Smith, Ina Smith, Alice Broos, Yu Hsin (Nina) Kung, Amanda McLaughlin, Anne Zeddeman

**Affiliations:** 1 Biosecurity Sciences Laboratory, Biosecurity Queensland, Department of Employment, Economic Development and Innovation, Brisbane, Queensland, Australia; 2 Australian Animal Health Laboratory, CSIRO Livestock Industries, East Geelong, Victoria, Australia; 3 Queensland Health Forensic and Scientific Services Laboratory, Queensland Health, Brisbane, Queensland, Australia; 4 University of Wageningen, Costerweg 50, Wageningen, Netherlands; Veterinary Laboratories Agency, United Kingdom

## Abstract

Hendra virus is a recently emerged zoonotic agent in Australia. Since first described in 1994, the virus has spilled from its wildlife reservoir (pteropid fruit bats, or ‘flying foxes’) on multiple occasions causing equine and human fatalities. We undertook a three-year longitudinal study to detect virus in the urine of free-living flying foxes (a putative route of excretion) to investigate Hendra virus infection dynamics. Pooled urine samples collected off plastic sheets placed beneath roosting flying foxes were screened for Hendra virus genome by quantitative RT-PCR, using a set of primers and probe derived from the matrix protein gene. A total of 1672 pooled urine samples from 67 sampling events was collected and tested between 1 July 2008 and 30 June 2011, with 25% of sampling events and 2.5% of urine samples yielding detections. The proportion of positive samples was statistically associated with year and location. The findings indicate that Hendra virus excretion occurs periodically rather than continuously, and in geographically disparate flying fox populations in the state of Queensland. The lack of any detection in the Northern Territory suggests prevalence may vary across the range of flying foxes in Australia. Finally, our findings suggest that flying foxes can excrete virus at any time of year, and that the apparent seasonal clustering of Hendra virus incidents in horses and associated humans (70% have occurred June to October) reflects factors other than the presence of virus. Identification of these factors will strengthen risk minimization strategies for horses and ultimately humans.

## Introduction

**Figure 1:**
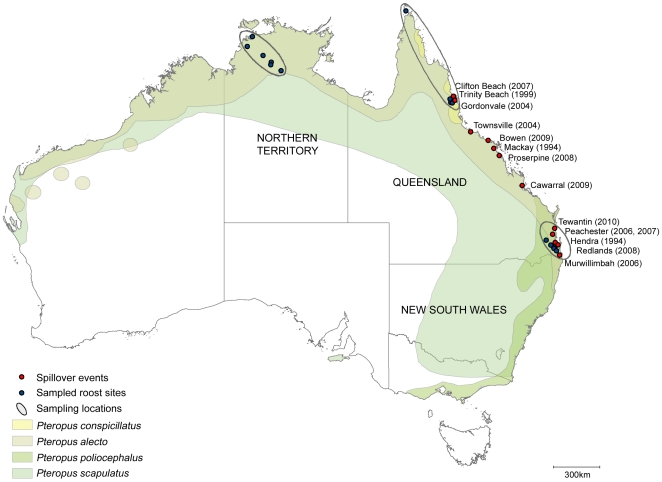
Map of Australia with flying fox distribution, Hendra virus spillover locations and flying fox sampling locations. Sampled roost sites were identified in three geographically disparate sampling locations: south-east Queensland (SEQ), far north Queensland (FNQ) and the Northern Territory (NT). The SEQ sampling location includes the sampled roost sites and spillover locations from Murwillimbah to Tewantin; the FNQ sampling location includes sampled roost sites and spillover locations from Gordonvale to Clifton Beach, and north to the tip of Queensland; the NT sampling location includes sampled roost sites identified in the Northern Territory, remote from any spillover locations.

Hendra virus is a sporadic, but highly lethal, recently emerged zoonotic agent in Australia. Case fatality rate in humans is 60%, and in horses, 75%. Since 1994, when it was first described, this novel member of the family *Paramyxoviridae* has spilled from its wildlife reservoir on 14 identified occasions (Figure 1), resulting in 45 attributed equine cases and 7 human cases [1], [2]. Fruit bats of the genus *Pteropus* (family *Pteropodidae*), colloquially known as flying foxes, are the natural host of the virus, and are asymptomatically infected [3]. Sero-epidemiologic studies have demonstrated evidence of infection in all four *Pteropus* species occurring on mainland Australia (*P. alecto*, *P. conspicillatus*, *P. poliocephalus*, *P. scapulatus*), and across their geographic range [3], [4]. However, difficulty in detecting virus (cf. antibodies) in flying foxes has previously limited understanding of infection prevalence and infection dynamics in the natural host, and constrained spillover risk management.

We undertook a three-year longitudinal study that used a quantitative RT-PCR assay to detect Hendra virus genome in pooled urine samples collected under flying fox roosts. The objectives of the study were two-fold: to investigate infection occurrence and frequency in flying fox populations, and to investigate the genetic diversity of Hendra viruses in flying fox populations. In this paper, we describe the former.

## Methods

### Ethics statement

Fieldwork was conducted under Department of Employment, Economic Development and Innovation Animal Ethics Committee permit SA 2008/10/270, Queensland Department of Environment and Resource Management Scientific Purposes permit WISP05810609, and the Northern Territory Parks and Wildlife Commission permit 37243.

### Field methods

Flying fox colonies rather than individual flying foxes were targeted. Multiple roost sites were identified in three geographically disparate sampling locations (>1500 km apart) in south-east Queensland (SEQ), far north Queensland (FNQ) and in the Northern Territory (NT) (Figure 1). Sampled roost sites included Canungra, Cedar Grove, Esk, Slacks Creek, Regents Park and Woodend (SEQ), Gordonvale, Lakeside, Mareeba, Thursday Island and Tolga Scrub (FNQ), and Howard Springs, Mataranka, Pine Creek, Katherine Gorge, Tindal RAAF Base and Wangi Falls (NT). Sampling locations were visited every 4–8 weeks (a sampling event) where possible, and an occupied roost site purposively selected. In the late afternoon, at the selected roost, plastic sheeting (typically measuring 3 m.×2 m.) was placed under trees in which flying foxes were roosting. The following morning at sunrise, pooled urine samples were collected from each sheet using a graduated micropipette (Soccorex ™) and 1 ml filter tip (Neptune ™),placed in a graduated 2 ml tube (Sarstedt microtube ™) and held on wet ice. The target sample number and volume was 30×2 ml samples (typically, three pooled samples from each of ten plastic sheets). Depending on roosting density, each sheet could hold urine from an estimated 5–20 individuals, giving a sample size of 50–200 individuals from ten sheets, and a minimum detectible infection prevalence of between 1.5 and 6% (with 95% statistical confidence).

After all samples were collected, 560 ul of urine was taken by graduated micropipette from each sample and added to an equal volume of lysis buffer (Buffer AVL from QIAGEN Viral RNA minikit ™) in a second 2 ml tube, to inactivate virus particles and preserve RNA. Then 500 ul of virus transport media (VTM) was added to the balance of the sample in the original tube. Samples were kept on wet ice or refrigerated until they could be packed and shipped on dry ice (according to IATA requirements, and typically within 48 hrs) to the Queensland Health Forensic and Scientific Services (QHFSS) laboratory (prior to March 2009) or the Biosecurity Sciences Laboratory (BSL) in Brisbane. At QHFSS/BSL, the samples were held in the PC3 (physical containment level 3) laboratory, the lysis buffer samples at 4°C pending RNA extraction and PCR analysis, and the VTM samples in a biosafety container at −80°C pending PCR results.

### Laboratory methods

At QHFSS/BSL, viral RNA was extracted using a QIAGEN viral RNA minikit™ and following a modified ‘large volume’ protocol [5] (Smith, C. *et al*, unpublished). The extracted eluate was screened for Hendra virus RNA by TaqMan™ RT-PCR using a set of primers and probe derived from the matrix protein gene of Hendra virus [6]. Eluates testing positive on the TaqMan™ assay, plus the corresponding VTM aliquot, were forwarded to the CSIRO Australian Animal Health Laboratory (AAHL) in Geelong, Australia for cell culture and virus isolation under PC4 conditions.

### Data management and analysis

Data were stored and managed in a Microsoft Office Access™ database. Analyses were performed on extracted data exported to a Microsoft Office Excel™ spreadsheet. P values of the chi square statistic are used to examine statistical association at the 95% confidence level. Where the nature of the data precludes the use of the chi square test, 95% binomial confidence intervals (calculated in Exact ™) are presented.

## Results

Table S1 presents details of all sampling events over the three year study period. A total of 1672 pooled urine samples from 67 sampling events was collected and tested between 1 July 2008 and 30 June 2011. Numbers varied with year and location, with 311 samples (19 sampling events) in year one, 465 samples (16 sampling events) in year two, and 896 samples (32 sampling events) in year three. There were 1138 samples (47 sampling events) from SEQ, 320 samples (12 sampling events) from FNQ and 214 samples (8 sampling events) from NT. Of the 67 sampling events, 8 were ‘follow-up’ events, prompted by previous detections, and are excluded from ‘prevalence’ analyses because these data are not independent (Table S1 and Table 1).

**Table 1:** Details of 20 sampling events^1^ yielding 45 positive pooled urine samples from July 2008 to June 2011.

Year	Sampling event date	Sampling location	Roost site	Species^2^ present	No. of samples collected	No. (%) of positive samples
Jul 08 to Jun 09	20-Nov	FNQ	Gordonvale	*P. consp.*	20	3 (15.0)
	12-Feb	SEQ	Slacks Creek	*P. alecto, P. polio. & P. scap.*	20	1 (5.0)
	12-Jun	SEQ	Slacks Creek	*P. alecto & P. polio.*	13	1 (7.7)
Jul 09 to Jun 10	5-Aug	SEQ	Cedar Grove	*P. alecto & P. polio.*	30	2 (6.7)
	24-Aug	FNQ	Tolga Scrub	*P. consp. & P. scap.*	30	2 (6.7)
	25-Sep	SEQ	Cedar Grove	*P. alecto & P. polio.*	36	12 (33.3)
	2-Oct	SEQ	Cedar Grove	*P. alecto & P. polio.*	45	2 (4.4)
	9-Oct^1^	SEQ	Cedar Grove	*P. alecto & P. polio.*	17	2 (11.8)
	20-Oct^1^	SEQ	Cedar Grove	*P. alecto & P. polio.*	32	4 (12.5)
	22-Oct	FNQ	Lakeside	*P. consp.*	35	2 (5.7)
	25-Mar	SEQ	Cedar Grove	*P. alecto & P. scap.*	34	1 (2.9)
Jul 10 to Jun 11	6-Jul	FNQ	Mareeba	*P. consp.*	30	2 (6.7)
	5-Jan	SEQ	Cedar Grove	*P. alecto*	30	1 (3.3)
	1-Feb	SEQ	Cedar Grove	*P. alecto & P. polio.*	31	2 (6.5)
	10-Feb^1^	SEQ	Cedar Grove	*P. alecto & P. polio.*	32	1 (3.1)
	24-Feb^1^	SEQ	Cedar Grove	*P. alecto & P. polio.*	24	1 (4.2)
	25-Mar^1^	SEQ	Cedar Grove	*P. alecto & P. polio.*	28	1 (3.6)
	12-May	SEQ	Cedar Grove	*P. alecto*	20	2 (10.0)
	28-May	FNQ	Lakeside	*P. consp.*	30	2 (6.7)
	3-Jun	SEQ	Tewantin	*P. alecto & P. polio.*	13	1 (7.7)

1 Five of the 20 sampling events yielding positive samples were ‘follow-up’ events prompted by previous detections. They are presented here for completeness, but excluded from ‘prevalence’ analyses as these data are not independent.

2 *P. polio.*, *P. consp.* and *P. scap.* are abbreviations of *Pteropus poliocephalus*, *P. conspicillatus* and *P. scapulatus* respectively.

Table1 presents details of all sampling events yielding positive urine samples. Figure 1 illustrates the geographic distribution of the four species of flying fox occurring on mainland Australia, the locations and dates of the 14 identified spillover events to horses, and the flying fox sampling locations and sampled roost sites.

Over the three-year study period, 25% (15/59) of sampling events yielded detections. There was no statistically significant association with year or location, the proportion of detections being 15.8% (3/19) in year one, 42.9% (6/14) in year two, and 23% (6/26) in year three (p = 0.20), and 26% (10/39) in SEQ, 41.7% (5/12) in FNQ, and 0% (0/8) in NT (p = 0.11). Table 2 presents a collapsed within-year summary of sampling events by month, and shows that positive sampling events occurred in all months except April (0/8) and December (0/4); the overlapping 95% confidence intervals suggest no statistically significant difference between months.

**Table 2:** The within-year distribution of 15 positive sampling events^1^ yielding 36 positive pooled urine samples from July 2008 to June 2011.

Month	No. of sampling events^1^	No. of positive sampling events^1^	% (95% CI) positive sampling events	No. of samples from positive sampling events	No. of positive samples from positive sampling events	% (95% CI) positive samples from positive sampling events
**Jan**	1	1	100 (2.5–100)	30	1	3.3 (0.1–17.2)
**Feb**	5	2	40 (5.3–85.3)	51	3	5.8 (1.2–16.2)
**Mar**	7	1	14.3 (0.4–57.9)	34	1	2.9 (0.1–15.3)
**Apr**	8	0	0 (0–36.9)	0	0	0
**May**	3	2	66.7 (9.4–99.2)	50	4	8 (2.2–19.2)
**Jun**	5	2	40 (5.3–85.3)	26	2	7.7 (0.1–25.1)
**Jul**	6	1	16.7 (0.4–64.1)	30	2	6.7 (0.1–22.1)
**Aug**	5	2	40 (5.3–85.3)	60	4	6.7 (1.8–16.2)
**Sep**	5	1	20 (0.5–71.6)	36	12	33.3 (18.6–51.0)
**Oct**	5	2	40 (5.3–85.3)	80	4	5 (1.4–12.3)
**Nov**	5	1	20 (0.5–71.6)	20	3	15 (3.2–37.9)
**Dec**	4	0	0 (0–60.2)	0	0	0

1 Five sampling events yielding positive samples (and three sampling events yielding negative samples) were ‘follow-up’ events prompted by previous detections, and are excluded as these data are not independent.

Of the 1460 pooled urine samples collected within the 59 sampling events, 2.5% (36/1460) yielded detections. The proportion of positive samples was statistically associated with both year and location, being 1.6% (5/311) in year one, 5% (21/416) in year two, and 1.4% (10/733) in year three (p = 0.0003), and 2.7% (25/926) in SEQ, 3.44% (11/320) in FNQ, and 0% (0/214) in NT (p = 0.03). In the collapsed within-year analysis (Table 2), the non-overlapping 95% confidence intervals indicate that the proportion of positive samples is statistically significantly higher in September (33.3%, 95% CI 18.6–51%) than in January, February, March, August and October.

Infection was detected in mixed species roosts (*P. alecto* and *P. polioceplalus*; *P. alecto* and *P. scapulatus*; *P. conspicillatus* and *P. scapulatus*; *P. alecto*, *P. polioceplalus* and *P. scapulatus*) and single species roosts (*P. alecto*, *P. conspicillatus*) (Table 1).

## Discussion

While serologic surveys have provided evidence of previous Hendra virus infection in Australian flying fox populations, successful attempts to demonstrate current infection have been limited [7]. For this study, we implemented a sampling and testing protocol that sought to maximize the likelihood of detection if infection was present. Firstly, we collected pooled urine samples from under flying fox colonies, as had Chua, 2003 [8], rather than catching and sampling individual animals, so each sample potentially represented multiple individuals. Flying foxes forage nocturnally, and roost communally in trees during the day, in colonies ranging from tens to hundreds of thousands of individuals. When they return to roost pre-dawn, they frequently urinate, and the urine is readily caught on strategically placed plastic sheeting. This approach greatly increased the number of animals sampled, and thus the efficiency of sample collection. Secondly, influenced by the success of Walcharaplusadee *et al* (2005) [5], we collected and extracted from a large volume of urine, increasing the likelihood of extracting RNA (or more RNA) if virus was present. Thirdly, we screened for extracted RNA using a set of primers and probe derived from the matrix protein gene of Hendra virus [6], as this gene appears to be more highly conserved, hence this assay was more likely to detect any Hendra virus variants. The approach yielded the first ever identification of Hendra virus genome in the urine of naturally infected free-living flying foxes, within the first five months of the study.

We believe that our 1.5–6% minimum detectable prevalence range, based on a typical 30 pooled urine samples from 10 sheets (and representing 5–20 individuals per sheet) is conservative; that is, we may sometimes be detecting infection present at lower prevalence. To elaborate, the figure of 5 individuals per sheet reflected the typical number of *P. alecto*, *P. conspicillatus* or *P. poliocephalus* that (from our extensive observations) would roost within a 3 m.×2 m. perimeter (the dimensions of each sheet); the figure of 20 individuals per sheet reflected the typical number of the more densely roosting *P. scapulatus* that would roost within the same perimeter. However the calculations do not consider the often multiple vertical ‘layers’ of flying foxes above sheets that could result in two or three times as many individuals over sheets, significantly lowering the minimum prevalence detectable by 30 pooled samples.

The number of sampling events per annum over the three-year study ranged from 16 to 32, the latter primarily reflecting an increased effort on FNQ and NT samplings in the third year. In all three years, the majority of the sampling events were in SEQ (14/19, 14/16 and 19/32 respectively), due primarily to logistical constraints to sampling in FNQ and NT. However, in the third year, we were able to increase the number of FNQ and NT samplings to reach our target sampling frequency (every 7–8 weeks) in these locations. We also sought to increase our sampling efforts at all locations in January and February in the third year (these months coincide with the peak of the wet season in Queensland), and were successful in SEQ, but not in FNQ and NT (because of an extended, and at times extreme, wet season in 2011). The number of SEQ samplings stayed relatively constant (14, 14, 19) across the three years, reaching or exceeding our target sampling frequency (every 4 weeks) for this location. The increased number of SEQ sampling events in the third year reflected a period of ‘follow-up’ sampling from January to early-April 2011, to gather additional information on infection dynamics in a colony in which infection was detected. A similar period of increased sampling frequency also occurred August to October 2009 in the same colony following detection of infection. Comparison of the two periods reveals contrasting infection dynamics, with the 2009 period indicating an explosive outbreak (peaking with 33.3% of urine samples positive), and the 2011 period suggesting a smoldering infection (Table S1). While the data should not be over-interpreted, these two dynamics are theoretically consistent with contrasting levels of ‘herd immunity’ in the colony on these two occasions, the former suggesting a high proportion of susceptible individuals, the latter suggesting a low proportion of susceptible individuals. Interestingly, Plowright *et al* (2011) [9] concluded from modeling simulations that both explosive and smoldering epidemics are necessary for the long-term persistence of Hendra virus in nature.

The proportion of positive sampling events and the proportion of positive samples provide two distinct measures of ‘prevalence’, the former between sampling events, and the latter within sampling events. We found that positive events were not significantly associated with location or year, whereas positive samples were. The wide 95% confidence intervals suggest the lack of association with sampling event (given the trend suggested by the point estimates) is primarily a reflection of the (statistically) limited number of sampling events, rather than a true absence of association. Because multiple samples were collected in each sampling event, sample size does not constrain statistical confidence, and we see a strong association between positive samples and both variables. Firstly considering location, we found that the proportion of positive samples was similar in SEQ and FNQ over the study period, and statistically different from NT, where no samples were positive. The proportion of positive sampling events showed the same pattern, but was not statistically significantly different. This finding suggests a real difference in the prevalence of Hendra virus infection in flying fox populations in Queensland and the Northern Territory. Whether this difference truly reflects location, or is confounded by species, population structure or some other variable cannot be ascertained from our study. Nonetheless, the finding of different infection prevalence in flying fox populations in different locations (for whatever reason) suggests a rationale for the (to date) geographic predominance of Hendra spillover events in Queensland, when flying foxes and horses exist in similar overlapping densities elsewhere in Australia.

Considering detections over time, we found that the proportion of positive samples was significantly higher in year two than in years one and three, indicating between-year variation in Hendra virus infection prevalence in flying fox populations. This finding fits well with the observed variable annual frequency of equine cases of Hendra virus, and suggests that ecological, physiological or other factors moderate infection or excretion in flying foxes.

We were interested to explore the ‘seasonal’ pattern of virus excretion from flying foxes to compare and contrast with the cumulative within-year occurrence of equine cases. When we collapsed the three years of data into a single 12-month period, we found positive sampling events in all months except April and December, and while statistical confidence was lacking (again evidently due to the modest number of sampling events), the over-lapping 95% confidence intervals suggest detections can occur in any month. To examine whether more detections might occur in some months, we sought an association between the proportion of positive samples and month. While the derived 95% confidence interval for September suggests at least a partial association, the collapsed September data actually represents a single positive sampling event in 2009 in which 33.3% of samples were positive (discussed above), thus this analysis should not be over-interpreted. Our finding of positive sampling events throughout the year is consistent with those of Walcharaplusadee *et al* (2005) [10] and Epstein *et al* (2011) [11] in relation to Nipah virus detections in flying foxes in Thailand and Bangladesh respectively. Significantly, our findings indicate that flying foxes can be infected and excrete virus at any time of year, and that the apparent clustering of Hendra virus incidents in horses and associated humans (70% of recognised incidents have been between June and October) reflects factors other than the presence of virus. Identification of these factors will strengthen risk minimization strategies for horses and ultimately humans.

The detection of positive samples from single species roosts of *P. alecto* and *P. conspicillatus* demonstrates that Hendra virus is currently cycling in populations of these species. Where positive samples were obtained from mixed species roosts, it was not possible to conclusively attribute collected urine to a particular species. Previous serologic surveys have found anti-Hendra virus antibodies in all four species [3], [4], but these findings do not provide a robust insight into current infection status, nor to whether some species are more ‘efficient’ natural hosts, in terms of infection prevalence or excreted viral load. This subject warrants further investigation, as it may provide an insight into the ‘Queensland-centric’ pattern of spillover events to date.

Our findings indicate that Hendra virus is not present in all flying fox colonies all of the time, and that the level of excretion in any particular colony fluctuates over time. In this study, 8.5% of samples from positive sampling events were positive, with a range of 3 to 33%, again indicating the dynamic nature of infection in any particular colony. These figures may be an over-estimate of the true population prevalence, given the possibility that multiple positive (pooled) samples on individual sheets may reflect a single individual. Paradoxically, while the data indicate that infection in any given flying fox colony is evidently periodic and transient, from a risk management perspective, it is appropriate to assume that any colony could be infected at any time, and for horse-owners, veterinarians and para-veterinarians to adopt recommended exposure risk minimization strategies [12].

Screening populations rather than individuals, and seeking to maximize the likelihood of detection at each step has been an effective approach to investigating Hendra virus infection dynamics in flying fox populations, and our study has yielded a number of significant findings. Firstly, Hendra virus excretion occurs periodically, rather than continuously, and in geographically disparate populations in Queensland. Secondly, the lack of any detection in the Northern Territory suggests prevalence may be higher in Queensland than elsewhere. A proposed expanded comparative prevalence study between Queensland, the adjacent state of New South Wales, and the Northern Territory aims to clarify this. Additional years of data are needed in the current study to improve the robustness of statistical comparisons within-year and between-year, and a further three years are planned. Finally, the finding that detections in flying foxes did not temporally cluster with recorded spillover events to horses was illuminating, and suggests a ‘necessary and sufficient cause’ paradigm for Hendra virus spillovers. That is, for spillover to horses to occur, not only must virus be present, but also must one or more ‘pre-disposing’ factors that increase the likelihood of infection. Investigating such plausible host, agent or environmental factors was beyond the scope of this study, and underlines the need for on-going longitudinal studies.

### Addendum

Since submission of the manuscript, the occurrence of an unprecedented cluster of incidents in Queensland and News South Wales (more than doubling the previously known number) dramatically illustrates our finding of between-year variation in virus excretion, and reinforces our key conclusions and recommendations, particularly the ‘necessary and sufficient cause’ paradigm, and the proposed expanded research on infection and transmission dynamics in Queensland and New South Wales.

## Supporting Information

Table S1 **Details of the total 67 sampling events yielding 1672 pooled urine samples from July 2008 to June 2011.**
(DOC)
