# Supplementary material for: Hendra Virus Infection Dynamics in Australian Fruit Bats
Source: PLoS One. 2011 Dec 9;6(12):e28678. doi: 10.1371/journal.pone.0028678 (PMC3235146; doi:10.1371/journal.pone.0028678)
Supplement: Table S1 — Details of the total 67 sampling events yielding 1672 pooled urine samples from July 2008 to June 2011. (DOC) [file pone.0028678.s001.doc]

| Table S1: Details of 67 sampling events yielding 1672 pooled urine samples from July 2008 to June 2011. | | | | | | |
| --- | --- | --- | --- | --- | --- | --- |
| **Year** | **Sampling Event^1^ Date** | **Sampling Location** | **Roost Site** | **Species^2^ Present** | **No. of**  **Pooled Samples Collected** | **No. (%) of Positive Pools** |
| Jul 08 to Jun 09 | 30-Jul | SEQ | Regents Park | *P. alecto & P. polio.* | 20 |  |
|  | 8-Aug | SEQ | Slacks Creek | *P. alecto & P. polio.* | 18 |  |
|  | 17-Sep | SEQ | Slacks Creek | *P. polio.* | 4 |  |
|  | **20-Nov** | **FNQ** | **Gordonvale** | ***P. consp.*** | **20** | **3 (15.0)** |
|  | 28-Nov | SEQ | Slacks Creek | *P. alecto, P. polio. & P. scap.* | 6 |  |
|  | 16-Dec | NT | Pine Creek & Mataranka | *P. alecto & P. scap.* | 17 |  |
|  | 18-Dec | FNQ | Gordonvale | *P. consp.* | 21 |  |
|  | 6-Feb | SEQ | Cedar Grove | *P. alecto* | 20 |  |
|  | **12-Feb** | **SEQ** | **Slacks Creek** | ***P. alecto, P. polio. & P. scap.*** | **20** | **1 (5.0)** |
|  | 25-Feb | SEQ | Esk | *P. alecto, P. polio. & P. scap.* | 21 |  |
|  | 6-Mar | SEQ | Slacks Creek | *P. alecto, P. polio. & P. scap.* | 18 |  |
|  | 24-Mar | SEQ | Canungra | *P. alecto & P. polio.* | 20 |  |
|  | 25-Mar | SEQ | Slacks Creek | *P. alecto & P. polio.* | 15 |  |
|  | 26-Mar | SEQ | Esk | *P. alecto & P. polio.* | 18 |  |
|  | 27-Mar | FNQ | Thursday Island | *P. alecto* | 14 |  |
|  | 9-Apr | SEQ | Canungra | *P. polio.* | 20 |  |
|  | 17-Apr | SEQ | Slacks Creek | *P. alecto & P. polio.* | 13 |  |
|  | **12-Jun** | **SEQ** | **Slacks Creek** | ***P. alecto & P. polio.*** | **13** | **1 (7.7%)** |
|  | 23-Jun | FNQ | Tolga Scrub | *P. consp.* | 13 |  |
| Jul 09 to Jun 10 | 7-Jul | SEQ | Slacks Creek | *P. alecto & P. polio.* | 18 |  |
|  | 15-Jul | SEQ | Slacks Creek | *P. alecto & P. polio.* | 13 |  |
|  | **5-Aug** | **SEQ** | **Cedar Grove** | ***P. alecto & P. polio.*** | **30** | **2 (6.7)** |
|  | **24-Aug** | **FNQ** | **Tolga Scrub** | ***P. consp. & P. scap.*** | **30** | **2 (6.7)** |
|  | **25-Sep** | **SEQ** | **Cedar Grove** | ***P. alecto & P. polio.*** | **36** | **12 (33.3)** |
|  | **2-Oct** | **SEQ** | **Cedar Grove** | ***P. alecto & P. polio.*** | **45** | **2 (4.4)** |
|  | **9-Oct^3^** | **SEQ** | **Cedar Grove** | ***P. alecto & P. polio.*** | **17** | **2 (11.8)** |
|  | **20-Oct^3^** | **SEQ** | **Cedar Grove** | ***P. alecto & P. polio.*** | **32** | **4 (12.5)** |
|  | **22-Oct** | **FNQ** | **Lakeside (Yungaburra)** | ***P. consp.*** | **35** | **2 (5.7)** |
|  | 8-Dec | SEQ | Cedar Grove | *P. alecto* | 30 |  |
|  | 19-Feb | SEQ | Cedar Grove | *P. alecto & P. scap.* | 28 |  |
|  | **25-Mar** | **SEQ** | **Cedar Grove** | ***P. alecto & P. scap.*** | **34** | **1 (2.9)** |
|  | 7-Apr | SEQ | Cedar Grove | *P. alecto* | 30 |  |
|  | 30-Apr | SEQ | Cedar Grove | *P. alecto & P. polio.* | 30 |  |
|  | 10-Jun | SEQ | Regents Park | *P. alecto & P. polio.* | 23 |  |
|  | 25-Jun | SEQ | Woodend | *P. alecto & P. polio.* | 34 |  |
| Jul 10 to Jun 11 | **6-Jul** | **FNQ** | **Mareeba (Jack Bethel Park)** | ***P. consp.*** | **30** | **2 (6.7)** |
|  | 8-Jul | NT | Howard Springs | *P. alecto* | 23 |  |
|  | 30-Jul | SEQ | Woodend | *P. alecto & P. polio.* | 32 |  |
|  | 15-Aug | FNQ | Mareeba (Jack Bethel Park) | *P. consp. & P. scap.* | 33 |  |
|  | 31-Aug | SEQ | Cedar Grove | *P. alecto & P. polio.* | 22 |  |
|  | 8-Sep | SEQ | Cedar Grove | *P. alecto & P. polio.* | 31 |  |
|  | 16-Sep | FNQ | Lakeside (Yungaburra) | *P. consp.* | 32 |  |
|  | 19-Sep | NT | Litchfield NP (Wangi Falls) | *P. alecto* | 25 |  |
|  | 6-Oct | SEQ | Cedar Grove | *P. alecto & P. polio.* | 28 |  |
|  | 23-Oct | NT | Mataranka Thermal Pools | *P. scap.* | 34 |  |
|  | 27-Oct | FNQ | Lakeside (Yungaburra) | *P. consp.* | 32 |  |
|  | 10-Nov | SEQ | Cedar Grove | *P. alecto & P. polio.* | 32 |  |
|  | 11-Nov | SEQ | Woodend | *P. alecto, P. polio. & P. scap.* | 34 |  |
|  | 27-Nov | NT | Pine Creek | *P. alecto & P. scap.* | 35 |  |
|  | 1-Dec | SEQ | Cedar Grove | *P. alecto & P. polio.* | 24 |  |
|  | **5-Jan** | **SEQ** | **Cedar Grove** | ***P. alecto*** | **30** | **1 (3.3)** |
|  | 18-Jan**^3^** | SEQ | Cedar Grove | *P. alecto & P. scap.* | 30 |  |
|  | **1-Feb** | **SEQ** | **Cedar Grove** | ***P. alecto & P. polio.*** | **31** | **2 (6.5)** |
|  | **10-Feb^3^** | **SEQ** | **Cedar Grove** | ***P. alecto & P. polio.*** | **32** | **1 (3.1)** |
|  | 18-Feb**^3^** | SEQ | Cedar Grove | *P. alecto & P. polio.* | 31 |  |
|  | **24-Feb^3^** | **SEQ** | **Cedar Grove** | ***P. alecto & P. polio.*** | **24** | **1 (4.2)** |
|  | 10-Mar | SEQ | Cedar Grove | *P. alecto & P. polio.* | 30 |  |
|  | **25-Mar^3^** | **SEQ** | **Cedar Grove** | ***P. alecto & P. polio.*** | **28** | **1 (3.6)** |
|  | 1-Apr | SEQ | Cedar Grove | *P. alecto & P. polio.* | 22 |  |
|  | 8-Apr**^3^** | SEQ | Cedar Grove | *P. alecto* | 18 |  |
|  | 13-Apr | NT | Pine Creek | *P. alecto* | 28 |  |
|  | 14-Apr | NT | Katherine Gorge | *P. scap.* | 26 |  |
|  | 16-Apr | FNQ | Lakeside (Yungaburra) | *P. consp.* | 30 |  |
|  | **12-May** | **SEQ** | **Cedar Grove** | ***P. alecto*** | **20** | **2 (10.0)** |
|  | 25-May | NT | Tindal RAAF Base | *P. alecto* | 26 |  |
|  | **28-May** | **FNQ** | **Lakeside (Yungaburra)** | ***P. consp.*** | **30** | **2 (6.7)** |
|  | **3-Jun** | **SEQ** | **Tewantin** | ***P. alecto & P. polio.*** | **13** | **1 (7.7)** |
|  |  |  |  |  |  |  |
|  |  |  |  |  | 1672 | 45 (2.7%) |
| ^1^ Positive sampling events are **bolded**.  ^2^ *P. polio., P. consp.* and *P. scap.* are abbreviations of *Pteropus poliocephalus, P. conspicillatus* and *P. scapulatus* respectively.  Two periods of intensive follow-up sampling (Aug-Oct 2009 and Jan-Apr 2011), including 8 sampling events^3^ triggered by previous positive events (and excluded from analyses) are shaded. | | | | | | |
